# Feasibility of physical map construction from fingerprinted bacterial artificial chromosome libraries of polyploid plant species

**DOI:** 10.1186/1471-2164-11-122

**Published:** 2010-02-19

**Authors:** Ming-Cheng Luo, Yaqin Ma, Frank M You, Olin D Anderson, David Kopecký, Hana Šimková, Jan Šafář, Jaroslav Doležel, Bikram Gill, Patrick E McGuire, Jan Dvorak

**Affiliations:** 1 Department of Plant Sciences, University of California, Davis, CA 95616, USA; 2 Genomics and Gene Discovery Unit, Western Regional Research Center, USDA/ARS, 800 Buchanan Street, Albany, CA 94710, USA; 3 Laboratory of Molecular Cytogenetics and Cytometry, Institute of Experimental Botany, Sokolovská 6, CZ-77200 Olomouc, Czech Republic; 4 Department of Plant Pathology, Kansas State University, Manhattan, KS 66506, USA

## Abstract

**Background:**

The presence of closely related genomes in polyploid species makes the assembly of total genomic sequence from shotgun sequence reads produced by the current sequencing platforms exceedingly difficult, if not impossible. Genomes of polyploid species could be sequenced following the ordered-clone sequencing approach employing contigs of bacterial artificial chromosome (BAC) clones and BAC-based physical maps. Although BAC contigs can currently be constructed for virtually any diploid organism with the SNaPshot high-information-content-fingerprinting (HICF) technology, it is currently unknown if this is also true for polyploid species. It is possible that BAC clones from orthologous regions of homoeologous chromosomes would share numerous restriction fragments and be therefore included into common contigs. Because of this and other concerns, physical mapping utilizing the SNaPshot HICF of BAC libraries of polyploid species has not been pursued and the possibility of doing so has not been assessed. The sole exception has been in common wheat, an allohexaploid in which it is possible to construct single-chromosome or single-chromosome-arm BAC libraries from DNA of flow-sorted chromosomes and bypass the obstacles created by polyploidy.

**Results:**

The potential of the SNaPshot HICF technology for physical mapping of polyploid plants utilizing global BAC libraries was evaluated by assembling contigs of fingerprinted clones in an *in silico *merged BAC library composed of single-chromosome libraries of two wheat homoeologous chromosome arms, 3AS and 3DS, and complete chromosome 3B. Because the chromosome arm origin of each clone was known, it was possible to estimate the fidelity of contig assembly. On average 97.78% or more clones, depending on the library, were from a single chromosome arm. A large portion of the remaining clones was shown to be library contamination from other chromosomes, a feature that is unavoidable during the construction of single-chromosome BAC libraries.

**Conclusions:**

The negligibly low level of incorporation of clones from homoeologous chromosome arms into a contig during contig assembly suggested that it is feasible to construct contigs and physical maps using global BAC libraries of wheat and almost certainly also of other plant polyploid species with genome sizes comparable to that of wheat. Because of the high purity of the resulting assembled contigs, they can be directly used for genome sequencing. It is currently unknown but possible that equally good BAC contigs can be also constructed for polyploid species containing smaller, more gene-rich genomes.

## Background

Plant and animal genomes are currently sequenced either by a global shotgun sequencing approach [1] or by sequencing of large-insert genomic clones and assembling the global genome sequence from them (ordered-clone approach) [2]. The former approach is inherently faster and more economical since the entire genome sequence is generated in a single operation. To assemble a genome sequence, it is necessary to identify overlaps of individual reads among vast numbers of other reads. The presence of repeated sequences among the reads makes this task challenging in some genomes. This aspect of genome architecture is greatly exacerbated in plants with large genomes by the precipitous turnover of repeated sequences in the intergenic spaces. For instance, in the tribe Triticeae of the grass family, in which the sizes of genomes in diploid species range from 3.3 to 8.1 Gbp (reviewed in [3]), sequences filling the intergenic space are almost entirely replaced in about 3 million years, which is a turnover rate orders of magnitude faster than in primate genomes [4]. Because of large genome size and fast turnover rate of repeated sequences, the Triticeae genomes contain large numbers of very similar nucleotide sequences, which has precluded the use of the shotgun genome sequencing approach for diploid Triticeae species.

A special challenge presented to genome sequencing in plants is polyploidy. A large percentage of seed plants are polyploid [5]. Probably all plants are ancient polyploids (paleopolyploids) but since paleopolyploidy does not usually complicate genome sequencing, paleopolyploidy is not considered in this study. Plant polyploids are categorized as either autopolyploids with identical genomes or allopolyploids with related genomes that were contributed by different diploid species. A vast majority of plant polyploids are allopolyploids. The need to allocate sequence reads to respective genomes makes it exceedingly difficult to assemble global genome sequences of polyploid species from whole-genome shotgun sequence reads. For that reason, no polyploid plant genome has yet been sequenced by this approach.

The alternative approach, based on sequencing large-insert clones, potentially avoids the factors limiting the shotgun sequencing approach. The advent of the high-information-content-fingerprinting (HICF) of bacterial artificial chromosome (BAC) clones greatly increased fingerprinting throughput and fidelity [6-8]. With the five-color SNaPshot HICF technology [8], computer-driven fingerprint editing [9], contig assembly with the FPC program [10,11], and contig anchoring on high-resolution genetic maps with the highly multiplexed Illumina GoldenGate™ assays [12], it is now theoretically possible to construct physical maps for most diploid plants and animals, including ancient polyploids, such as maize and soybean [13,14].

The SNaPshot HICF fingerprinting technology is based on restriction digestion of the DNA of each BAC clone by multiple restriction endonucleases and sizing a portion of the fragments with capillary electrophoresis. Contigs are then assembled on the basis of shared portions of the restriction profiles of the BAC clones. It has been tacitly assumed that BAC clones from homoeologous chromosome regions in an allopolyploid will have too many restriction fragments in common and will be included into single contigs during contig assembly. Consequently, physical mapping based on the SNaPshot HICF technology has not been pursued to any significant extent in recently evolved allopolyploids, with the sole exception of hexaploid wheat, *Triticum aestivum*.

Polyploid wheat species of economical importance are either allotetraploid (*T. turgidum*, genome formula AABB) or allohexaploid (*T. aestivum*, genome formula AABBDD). The A, B, and D genomes were contributed by three different diploid species which radiated from a common ancestor between 2.5 and 4.5 million years ago, depending on which of several estimates is used, and are approximately equally diverged from each other at the molecular level [15,16]. Because of the recent divergence of the three ancestors, it was assumed that the assembly of contigs from a global *T. aestivum *BAC library would not produce physical maps of wheat chromosomes that would be of adequate quality for genome sequencing. Instead, technological advances in flow-sorting of chromosomes and the unique availability of individual chromosome and chromosome arm genetic stocks for wheat suggested an alternative procedure for generating hexaploid wheat physical maps. Chromosome-specific or chromosome-arm-specific BAC libraries are being constructed from DNA produced by flow-sorting of complete and telocentric chromosomes of *T. aestivum *[17] and used for the construction of the physical maps of the 21 *T. aestivum *chromosomes http://www.wheatgenome.org/. Such BAC libraries are easier to handle and simplify contig assembly compared to a global *T. aestivum *BAC library comprising over one million clones. Their availability also facilitates division of labor and international collaboration on the development of wheat sequence-ready physical maps. The successful construction of the physical map of *T. aestivum *chromosome 3B [18] from a chromosome 3B BAC library [17]demonstrated the feasibility of this approach.

For chromosome flow-sorting to be a general approach to produce physical maps of allopolyploid species, each chromosome in the karyotype of a targeted allopolyploid would have to be a unique size - a condition, which is seldom met. Alternatively, special cytogenetic stocks, such as telosomic lines and chromosome addition lines must be available for all chromosome arms present in the genome or developed *de novo *[19]. For these reasons, the physical mapping strategy adopted for sequencing of *T. aestivum *is not generally applicable, and genomes of most polyploid plants, including tetraploid wheat, cannot be physically mapped by this approach.

An assessment of the utility of the current HICF technology for the construction of physical maps of polyploid plants from global BAC libraries is therefore of central importance for advancing genome research on polyploid organisms. To date, because of the large costs involved, no relevant data on this subject exists. However, the ability to produce chromosome-specific physical maps in hexaploid wheat provides the opportunity to undertake the assessment using a single set of homoeologous chromosome arms. Such an assessment is reported here.

BAC libraries constructed from flow-sorted telocentric chromosomes 3AS and 3DS and complete chromosome 3B were employed. Telosomes 3AS and 3DS are homoeologous to each other and both are homoeologous to the short arm of chromosome 3B (arm 3BS). The three libraries were fingerprinted. Contigs were either assembled from the clones of a single library or fingerprints were merged, and contigs were assembled from the clones of the merged library. Since the origin of each clone in the merged library was known, the frequency of inclusion of clones from more than a single chromosome arm into the contigs could be quantified for the entire population of contigs.

## Methods

### BAC libraries

One chromosome-specific and two chromosome-arm specific BAC libraries from hexaploid wheat (*Triticum aestivum *L.) cv. 'Chinese Spring' were used in the present study (Table 1). All three libraries were constructed using the *Hin*dIII cloning site of the pIndigoBAC-5 vector from DNA of chromosomes and/or chromosome arms (telosomes) purified by flow cytometric sorting [17]. Although two 3AS-specific BAC libraries are currently available http://olomouc.ueb.cas.cz, only the TaaCsp3AShA library comprising 55,296 clones was used here [20]. Only one 3DS-specific BAC library (TaaCsp3DShA, [20]) is available at present and this library, comprising 36,864 clones, was used. Additionally, 3,840 BAC clones were randomly selected from the first chromosome 3B-specific BAC library (TaaCsp3BFhA, [17]). Of the 96,000 BAC clones 95,232 were fingerprinted at UC Davis using an identical fingerprinting procedure.

**Table 1:** Characteristics of the chromosome and chromosome arm BAC libraries used in the study

AC library	Chromosome	Chromosome size (Mbp)*	Average insert size	No. clones**	Contaminating clones***	Chromosome coverage
TaaCsp3AShA	3AS telosome	351	80 kb	55296	11.0%	12.6

TaaCsp3BFhA	3B	1044	103 kb	3840	11.4%	0.4

TaaCsp3DShA	3DS telosome	285	110 kb	36864	10.0%	14.2

*Chromosome molecular sizes were determined considering 1C genome size of wheat 16 937 Mbp [32] and relative chromosome lengths as reported by Dvorak at al. [28].

**The total number of clones in the supplied libraries.

***Contamination with other chromosomes

### SNaPshot HICF fingerprinting

The BAC clones of the 3AS, 3DS, and 3B BAC libraries were fingerprinted as described by Luo *et al*. [8] with minor modifications [21]. From each 384-well plate, four 96-well blocks containing 1.2 ml of 2× YT medium [22] were inoculated with cells with a 96-well replicator. Two pins were removed from the replicator for the insertion of control clones into the 96-well plate. Two control BAC clones were inserted manually in wells E07 and H12 in each 96-well block. The cultures were grown for 24 hours on an orbital shaker agitated at 400 rpm, 37C. BAC DNAs were isolated with the Qiagen R.E.A.L 96-Prep kit (Qiagen, Valencia, California). The following minor modifications of the fingerprinting method were made to accommodate the use of an ABI3730XL (Applied Biosystems, Foster City, California) instead of an ABI3100 for capillary electrophoresis. The more sensitive laser of the ABI3730XL instrument improved fingerprinting resolution and made it possible to reduce the amount of BAC DNA sample for electrophoresis, thus lowering fingerprinting costs. To reduce sample size, 0.5-1.2 μg instead of 1.0-2.0 μg of BAC DNA were simultaneously digested with 2.0 instead of 5.0 units each *Bam*HI, *Eco*RI, *Xba*I, *Xho*I, and *Hae*III (New England Biolabs, Beverly, Massachusetts) at 37C for 3 hrs. DNAs were labeled with 0.4 μl instead of 1.0 μl of the SNaPshot kit (Applied Biosystems, Foster City, California) at 65C for 1 hr and precipitated with ethanol. DNAs were dissolved in 9.9 μl of Hi-Di formamide, and 0.3 μl of Liz1200 size standard was added to each sample. Restriction fragments were sized on the ABI3730XL using 50 cm capillaries and POP7 (Applied Biosystems, Foster City, California). Fragment-size calling was accomplished with the GeneMaper software (Applied Biosystems, Foster City, California) with the help of FPPipeliner http://www.bioinforsoft.com/.

### Fingerprint editing

The GeneMaper output data were edited with the GenoProfiler program [9] and FPMiner http://www.bioinforsoft.com/. The control BAC clone 135H19 from barley Morex *Hin*dIII library.(.)[23] inserted in each 96-well plate was used to check for the correct orientation of the plate. Fingerprints of cross-contaminated samples were detected using a module in the GenoProfiler [9] and eliminated from the database. Data on the fragments in the size range 100 to 1000 bp were collected. The numbers of BAC clones used for contig assembly after editing are listed in Table 2.

**Table 2:** Numbers of BAC clones used for contig assembly and the numbers of assembled contigs and remaining singleton clones

Library used in contig assembly	No. clones remaining after editing	No. contigs	No. singletons
3AS	47063	1677	11939

3B	2973	562	1114

3DS	30517	1411	7843

Total	80553	3650	20896

Merged library		3369	23192

Contigs were assembled using a tolerance of 0.5 bp, starting with an initial Sulston score of 1 × 10^-50^. Contigs were deQed [11] until no contig contained more than 15% of Q clones. Singleton-to-contig joining was performed at Sulston scores of 1 × 10^-30 ^and 1 × 10^-22^. Remaining singletons were end-merged at Sulston scores of 1 × 10^-15^. Contigs were merged at Sulston scores of 1 × 10^-30^, 1 × 10^-20^, 1 × 10^-15^, and 1 × 10^-12^, requiring only a single clone overlap between contigs.

Fingerprinting of BAC clones from the 3AS, 3DS, and 3B libraries was performed using the same technique, by the same personnel, and using the same instruments sequentially during a time span of 12 months. To repeat the work and ascertain that data were not affected by a systemic difference that occurred over the 12-month period, 5,000 BAC clones each from the 3AS and 3DS libraries were re-fingerprinted side-by-side, i.e., equal numbers of 3AS and 3DS clones were included in each fingerprinting and fragment-sizing run. Contigs were assembled as described above.

### Fluorescence in situ hybridization (FISH) of BAC clones

Wheat root tips were maintained in ice water for 26 to 30 h and then fixed in a mixture of 3 parts of absolute ethanol: 1 part of glacial acetic acid at 37C for seven days. Cytological preparations and *in situ *hybridization with labeled DNA were made as described earlier [24]. DNAs of BAC clones were isolated and labeled with digoxigenin using the DIG-Nick Translation Kit or biotin-Nick Translation Kit (Roche Applied Science) according to manufacturer's recommendations. BAC-FISH was done as described by Nasoudi-Nejad et al. [24]. The site of probe hybridization was detected with the anti-DIG-FITC conjugate (Roche Applied Science) and by the streptavidin-Cy3 conjugate (Amersham, Piscataway, NJ, USA). For the identification of wheat genomes, metaphase figures were reprobed with two additional probes. Biotin-labeled probe was prepared using PCR with (GAA)_7 _and (CCT)_7 _primers and wheat genomic DNA as a template. This probe was used here to identify B-genome chromosomes. A probe for 260-bp fragment of the *Afa *family of repeats was prepared and labeled by digoxigenin using PCR with primers AS-A and AS-B on wheat genomic DNA as described earlier [25]. This probe was used here to identify D-genome chromosomes. Chromosomes were counterstained with 1.5 μg/ml 4',6-diamidino-2-phenylindole (DAPI) and observed with Olympus AX70 microscope with a SensiCam B/W CCD camera. Chromosome images were processed using the ScionImage and Adobe Photoshop v. 6 software.

## Results

### Contig assembly

Of 95232 clones fingerprinted, 80553 were suitable for assembly; the remaining clones were eliminated due to unsuitable length, contamination, and other reasons. Contigs were assembled for each library separately and for the three libraries merged into one. A total of 3650 contigs and 20896 singletons were obtained in the separate assemblies (Table 2). A total of 3369 contigs and 23192 singletons were obtained in contig assembly employing the merged library, which was close to the total numbers of contigs and singletons obtained in assemblies of individual libraries (Table 2).

To ascertain that no differences in the fingerprinting of the libraries took place, 1862 fingerprints of the control clone 135H19 inserted into each 96-well plate during the fingerprinting of the three libraries were subjected to assembly at a Sulston score of 1 × 10^-50^. If fingerprinting conditions of one library would differ from those used during fingerprinting of the other two libraries, the fingerprints of the control clone would be equally affected and would assemble into separate contigs. The assembly generated two contigs and 4 singletons. One contig contained 1855 fingerprints (99.62%) whereas the other contained 3 fingerprints (0.0015%). The latter contig was caused by the failure of size-standard electrophoresis. The four singletons had defective fingerprints. Disregarding the latter contig and the four singletons, all control fingerprints from the three libraries assembled into a single contig at very high assembly stringency. We conclude therefore that no difference during the fingerprinting and fragment sizing of the three libraries took place.

To repeat the experiment, 5000 clones from the 3AS and 3DS libraries each were re-fingerprinted together and fragments were sized (side-by-side fingerprinted clones). A total of 7881 clones were suitable for assembly. Contigs were assembled using stringency comparable to that used previously. A total of 1327 contigs and 3380 singletons were obtained.

### Contig analysis

Contigs generated by assembly of the 80553 clones in the merged library contained clones predominantly from single libraries (Table 3). The number of clones incorporated into wrong contigs represented only a small fraction of the total. The absolute numbers of incorporated clones were adjusted in terms of percentages for the differences in the sizes of the libraries to make the numbers comparable. The adjusted incorporation percentages of clones from a single library in a contig ranged from 97.78% for clones from the 3DS library to 99.66% for clones from the 3B library. Adjusted misincorporation percentages ranged from 0.64% to 2.22% of contig clones (Table 3).

**Table 3:** The numbers and sources of clones incorporated into contigs containing more than three clones during contig assembly of merged BAC library

	Clone library origin			
**Prevalent clones in a contig**	**3AS library**	**3B library**	**3DS library**	**Misincorporated clones (%)**

3AS	27423 (99.36%)	3 (0.22%)	143 (0.42%)	0.64%

3B	15 (0.17%)	578 (99.66%)	16 (0.17%)	0.34%

3DS	254 (1.43%)	7 (0.79%)	21956 (97.78%)	2.22%

Results obtained with side-by-side fingerprinting and fragment sizing of the 3AS and 3DS clones were similar. The vast majority of contigs contained only clones from single libraries (Table 4). The adjusted percentages of misincorporated clones were similar to those obtained previously for the 3AS and 3DS libraries. The reproducibility of the misincorporation percentages for the two libraries suggested that misincorporation of clones into contigs was not caused by biological factors but was an attribute of the libraries.

**Table 4:** Numbers of clones from each library incorporated into contigs containing two or more clones during contig assembly of re-fingerprinted 3AS and 3DS BAC libraries

	Clone library origin	
**Prevalent clones in a contig**	**3AS library**	**3DS library**

3AS	1860 (99.63%)	7 (0.37%)

3DS	40 (1.52%)	2594 (98.48%)

### Identity of misincorporated clones

**Figure 1:**
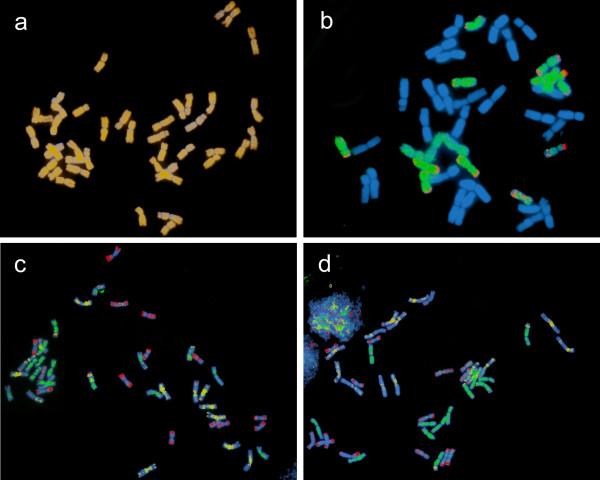
**BAC-FISH of selected BAC clones with the *T. aestivum *mitotic metaphase chromosomes**. (A) Clone 3AS0096P02 (labeled pale orange) showed a dispersed hybridization across all 21 pairs of chromosomes and could not be localized to a genome. (B) Clone 3AS0034G08 (labeled green) hybridized with 7 pairs of chromosomes belonging to the D genome identified using a probe for the *Afa *repeat family (labeled red). (C and D) Clones 3DS0002N13 (C) and 3DS0019H15 (D) (both labeled green) showed dispersed signal on 7 pairs of chromosomes belonging to the A genome, which were identified by the failure to hybridize with GAA microsatellites (labeled yellow) and an *Afa *family repeat (labeled red). All chromosomes were counterstained with DAPI (blue).

It was assumed to this point that all clones in a library were from the indicated chromosome. That assumption is unrealistic since DNA used for a library construction was generated by chromosome flow-sorting and was to some extent contaminated by fragments of other chromosomes (Table 1). Due to impurity of the libraries, a portion of clones in, e.g., the 3AS library, while labeled as 3AS clones, could actually come from other chromosome, including 3BS and 3DS, and could have been actually correctly incorporated into the 3BS and 3DS contigs, respectively, during merged library contig assembly. To determine if this possibility was real, BAC-FISH was done separately with ten randomly selected misincorporated BAC clones under various stringency conditions (77%, 87%, 93% and 98%). The stringency is the percentage of matches and mismatches between a probe and target nucleic acids that are allowed to occur without the double helix hybrid falling apart. BAC-FISH with seven of the clones produced dispersed signals over all 21 chromosome pairs of *T. aestivum *and provided no information (Fig. 1A). The 3AS-library BAC clone 3AS0034G08, misincorporated into a D-genome contig, produced signal along 7 pairs of the wheat chromosomes, indicating that it hybridized with the chromosomes of a single *T. aestivum *genome. Re-probing the same metaphase plate with the GAA satellite probe marking the B-genome chromosomes and the probe for *Afa *repeat marking the D-genome chromosomes showed that the BAC hybridized with the seven D-genome chromosome pairs and was actually a D-genome clone (Fig. 1B). Similarly, BAC clones 3DS0002N13 and 3DS0019H15 were 3DS-library clones that were misincorporated into A-genome contigs. BAC-FISH and FISH with the GAA and *Afa *repeat probes showed that these two clones hybridized with the seven A-genome chromosome pairs and were actually A-genome contaminants in the 3DS library (Fig. 1C and 1D).

It is expected that fingerprints of rare clones from other chromosomes contaminating a single-chromosome arm library would not overlap any clone in the library and would be left as singletons after contig assembly of individual libraries. Forty-six percent of clones that were misincorporated into 3B contigs were singletons in either the 3B-library assembly or the 3DS-library assembly; 84% of clones that were misincorporated into 3DS contigs were singletons in either the 3AS-library assembly or 3B-library assembly, but all clones that were misincorporated into 3AS contigs were incorporated into contigs during individual BAC library assemblies. These findings, except for the clones incorporated into the 3AS contigs, are consistent with the assumption that most of the misincorporated clones were clones from chromosomes contaminating the chromosome or chromosome-arm specific libraries.

## Discussion

### Mis-assembly of the merged library contigs

Contigs assembled from BAC clones of the merged library contained 97.78% (3DS contigs), 99.36% (3AS contigs), and 99.66% (3B contigs) clones from a single genome. This level of assembly fidelity is remarkable. Incorporation of less than 2.22% of clones in the contigs from homoeologous chromosomes would have no effect on assembly if sufficient genome coverage was used. In reality, the level of mis-assembly was much lower. BAC libraries produced from DNA isolated from flow-sorted chromosomes always show a certain level of contamination with clones from other chromosomes. The percentage of contaminating DNA was estimated to be from 10 to 11.4% in the three libraries (Table 1 and [17,20]). Assuming that the probability to contaminate a single chromosome arm library is equal for all remaining chromosome arms, it is expected that the libraries contained from 0.50% (10 × 2/40 arms) to 0.56% (11.4 × 2/41 arms) clones from the homoeologous chromosome arms. The observed percentages of misincorporated clones observed during contig assembly in the merged library were close to these numbers. To characterize a sample of misincorporated clones, BAC-FISH technique was selected from several possible techniques that could be used to determine genome origin of a clone [26,27]. BAC-FISH showed directly that some of the clones harbored DNA inserts from contaminating chromosomes during chromosome flow-sorting. The fact that most of the misincorporated clones were singletons during individual library assembly was consistent with the argument that misincorporated clones were in fact mostly contaminating clones. We therefore conclude that contigs generated by merged library assembly contained almost exclusively clones from only single wheat chromosome arms and the presence of BAC clones from homoeologous chromosome arms had no effects on the fidelity of contig assembly.

All three libraries used here were fingerprinted in the same lab, by the same workers, and sized on the same ABI3730XL DNA analyzers using the same size standard. Nevertheless, if fingerprinting or fragment sizing would drift over time, it is conceivable that the fingerprints of a library would be more similar to each other then to those of another library and would then assemble into separate contigs. Essentially all control clones inserted into fingerprinted plates assembled into a single contig, indicating that no difference in fingerprinting existed among the three libraries. Replication of the experiment using side-by-side fingerprinting and fragment sizing of a total of 10,000 BAC clones from the 3AS and 3DS libraries produced results nearly identical to those obtained with sequential fingerprinting of the libraries. We therefore conclude that no technical difference existed in the fingerprinting and fragment sizing of the three libraries.

It is hypothetically possible that unknown vector differences existed or pieces of foreign DNA were incorporated into clones during construction of libraries and resulted in preferential assembly of clones from a single library. The restriction fragments originating from these artifacts would be removed by the GenoProfiler software during BAC clone editing, in the same manner as the vector restriction fragments are removed [9], and would have no effect on contig assembly.

### Can physical maps of polyploid plants be constructed from global BAC libraries?

The ability to assemble contigs from a global BAC library of a polyploid species without amalgamating orthologous clones from homoeologous chromosomes into single contigs would open the door to the construction of physical maps of allopolyploid plants and greatly advance their genomic research. Results reported here showed that the contig assembly using BAC libraries consisting of clones of homoeologous chromosome arms 3AS, 3BS, and 3DS assembled contigs with negligible levels of contamination from homoeologous arms. Wheat chromosomes of homoeologous group 3 are the largest chromosomes in the respective wheat genomes and are nearly metacentric [28]. Their short arms therefore represent significant portions of the genomes and are therefore representative.

We conclude that the primary cause of the separate assembly of homoeologous contigs is the divergence of the intergenic spaces of homoeologous chromosomes. Intergenic spaces containing transposable elements account for large portions of wheat BAC clones and many clones consist only of TEs [29]. TEs in the intergenic spaces are subjected to a precipitous turnover rate in Triticeae genomes [4]. Sequencing of wheat A-, B- and D-genome BAC clones harboring orthologous genes showed that except for the gene(s) homoeologous clones had virtually no other sequences in common [4,30,31]. Restriction fragments generated by the digestion of genes account for a small portion of the total number of fragments in the fingerprint of wheat homoeologous BAC clones and have little effect on contig assembly.

There is no reason to assume that genome architecture in other allopolyploid plants with genomes of similar sizes to those of wheat differs from that found in wheat, and findings made here for wheat can likely be generalized to them. Whether the same contig assembly dynamics would prevail in allopolyploid species with small genomes, in which BAC clones would be inherently more gene-rich than in wheat, and hence share more fragments, is currently unknown and needs assessment.

## Conclusions

We conclude that SNaPshot HICF technology can be used to assemble BAC contigs and construct physical maps from whole-genome BAC libraries of allopolyploid species with genomes of comparable architecture to those of wheat. The minimum tiling path of clones across such contigs will represent single haplotypes and allow either shotgun sequencing of entire contigs or ordered-clone sequencing of individual clones, thus opening the door to genome sequencing of plant polyploid species.

## Authors' contributions

MCL, ODA, BSG, PEM and JDv designed the research; YM performed BAC DNA isolation and fingerprinting; YM and MCL performed fingerprint editing; MCL performed contig assembly; MCL, FMY and JDv performed contig analysis; JDo provided chromosome 3B BAC clones and revised the manuscript; HŠ and JŠ provided the 3AS and 3DS BAC libraries, DK performed the FISH experiments. MCL and JDv drafted the manuscript. All authors read and approved the final version of the manuscript.
